# Congenital Jejunal Web with Central Aperture in Children: Report of Two Cases of Delayed Diagnosis and Management

**DOI:** 10.1055/a-2839-6197

**Published:** 2026-04-02

**Authors:** Umama Huq, Md Hasanuzzaman, Sadruddin Al Masud, Kaniz Hasina, Zobaer Hassan Chowdhury

**Affiliations:** 1 Department of Paediatric Surgery, Dhaka Medical College and Hospital, Dhaka, Bangladesh

**Keywords:** jejunal web, congenital intestinal web, intestinal atresia

## Abstract

Congenital intestinal web of the jejunum is an exceptionally rare condition. While most intestinal atresias present during the neonatal period, jejunal webs with central apertures can lead to delayed presentations, often causing diagnostic challenges. We report two cases of jejunal web in children diagnosed beyond the neonatal period. Both patients presented with failure to thrive and bilious vomiting. One patient, a 2-year-3-month-old girl, was diagnosed with a single jejunal web and underwent successful web excision. The other, a 2-year-8-month-old girl, was found to have double jejunal webs—an extremely rare occurrence. She underwent resection and anastomosis but required reoperation due to anastomotic disruption. These cases highlight the diagnostic challenges associated with delayed presentation of jejunal webs and reinforce the importance of considering this rare anomaly in cases of chronic partial intestinal obstruction.

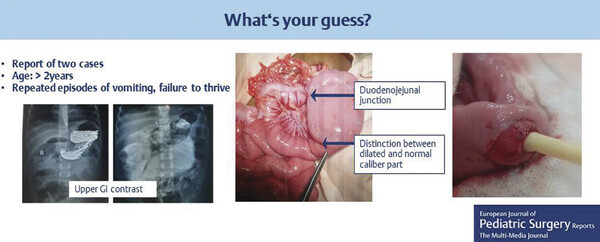

## Introduction

Congenital intestinal web or type I atresia or windsock deformity in the jejunum is a very rare entity. 1 The incidence of intestinal type I atresia ranges from 1 in 10,000 to 1 in 40,000 live births. 2 The majority of cases (85–90%) occur in the second portion of the duodenum, while involvement of the jejunum is exceedingly rare. 1 2 However, the exact incidence of type I jejuno-ileal atresia is unknown. 3 In a retrospective series of 37 patients with type I intestinal atresia, jejunal webs were identified in only 3 cases (8%). 4 Although most atresias present during the neonatal period, patients with this rare anomaly may present beyond the neonatal age due to the presence of a central aperture, leading to diagnostic dilemmas. 1 Unlike cases with complete atresia, these patients present with subtler features of chronic partial intestinal obstruction, such as failure to thrive, bilious vomiting, constipation, and abdominal distension. 5

We present two cases of jejunal web presenting after 2 years of age with failure to thrive and bilious vomiting. A recent review identified 18 reported cases of jejunal web with central fenestration presenting beyond the neonatal period, with ages ranging from 2.5 months to 50 years. 6 Additionally, a retrospective series reported two similar cases, making our patients the 21st and 22nd cases with such an entity in the English literature. 3 One of them was found to have double jejunal webs, constituting the third such case reported across all age groups and the second with delayed presentation (after the neonatal period). 7 8

## Case 1

A 2-year-3-month-old girl presented with bilious vomiting, constipation, and failure to thrive. Her weight was 6.3 kg, height 82 cm, falling below the third percentile (weight for height z score − 6.6 SD). The vomiting had started at 7 months of age and was occasional, bilious, and associated with difficult defecation. For 3 months before presentation, she experienced upper abdominal distension after meals, relieved by vomiting. The child's diet mostly contained khichuri (rice mixed with dal), seasonal vegetables, and fruits, along with breastfeeding. On clinical examination, the child appeared ill, with mild upper abdominal distension. She had been treated as a case of chronic constipation by local physicians for 1 year. She was prescribed lactulose and antiemetics. When the condition did not improve, the patient was referred to our center. Initial laboratory evaluation revealed Na ^+^ 130 mmol/L, K ^+^ 3.5 mmol/L, Cl ^−^ 98 mmol/L, and anemia (hemoglobin [Hb] 8 g/dL). An upper gastrointestinal series and small bowel follow-through revealed a grossly dilated stomach and duodenum.

**Fig. 1:**
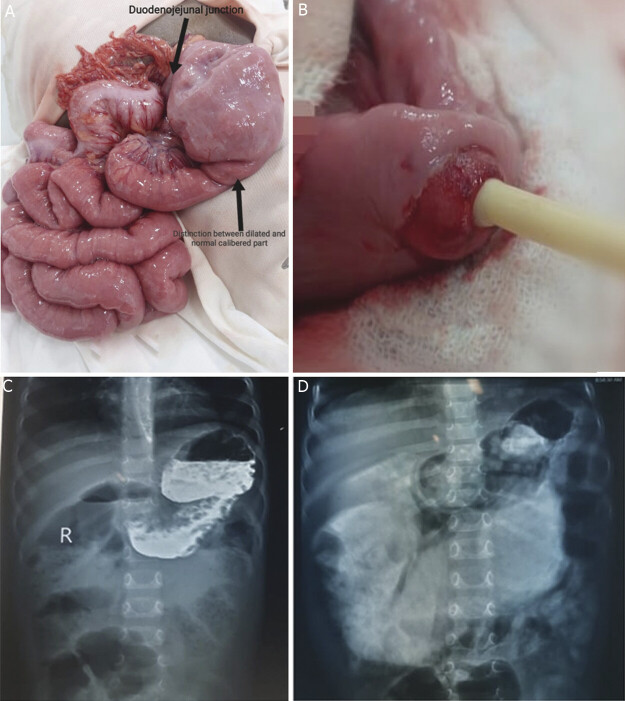
Panel ( **A** ) showing dilated jejunum and its clear distinction from normal caliber jejunum. Panel ( **B** ) demonstrates the jejunal membrane with the central hole. Panel ( **C** ) & ( **D** ) shows a contrast X-ray of a grossly dilated stomach with dilated upper gut.

Laparotomy was performed through a right supraumbilical transverse incision. A grossly dilated stomach, duodenum, and jejunum up to 15 cm from the ligament of Treitz were found, with a distinct transition between the dilated and normal caliber distal jejunum ( Fig. 1A ). An enterotomy was made on the antimesenteric border, proximal to the discrepancy. A Foley catheter was introduced distally, its balloon inflated and drawn back to delineate a membrane with a central hole ( Fig. 1B ). The membrane was excised, and the enterotomy was closed transversely after ensuring distal bowel patency with a saline test. Postoperative recovery was uneventful; bowel movement resumed within 2 days, oral feeding started on the postoperative day (POD) 4, and the patient was discharged on POD 6. At the 2-month follow-up visit, the patient's weight had increased to 7.5 kg, indicating satisfactory weight gain following surgical correction.

## Case 2

The second case involved a 2-year-8-month-old girl who presented with upper abdominal distension. Her mother reported frequent non-bilious vomiting over the past 1 year after starting solid food, which had turned bilious in the last 2 weeks. From 4 months of age, the child had been consuming only suji and cow's milk. Over the past year, she experienced multiple hospital admissions for vomiting and dehydration, without any significant physical examination findings. She was treated with intravenous fluid and omeprazole on each admission. On query, the mother also gave a history of visible peristalsis after feeding. She weighed only 7 kg with a height of 75 cm, both below the third percentile, and demonstrated features of failure to thrive. Her weight-for-height z-score was < − 3 SD, indicating severe wasting according to the WHO growth standards. Laboratory results showed albumin 2.5 g/dL, Hb 10 g/dL, and hyponatremia (Na ^+^ 127 mmol/L). Abdominal ultrasound revealed dilated bowel loops. An upper GI contrast study, done to rule out malrotation, showed a dilated stomach and duodenum ( Fig. 1C ).

After preoperative optimization, laparotomy revealed dilation of the upper gastrointestinal tract up to 30 cm from the ligament of Treitz. An enterotomy was made proximal to the obstruction, where a membrane with a central aperture was identified. A second membrane was found 5 cm distal to the first during the saline test, which was performed to rule out any other obstruction. As the proximal gut was hugely distended, the surgeon preferred an en bloc resection with end-to-end anastomosis. A portion of the dilated segment was resected, and owing to both the debated benefits of tapering jejunoplasty and the unavailability of a gastrointestinal stapler at our center, primary anastomosis was considered sufficient. Oral feeding was initiated on the POD 5; however, the patient subsequently developed bilious vomiting and abdominal distension. Plain abdominal radiograph revealed no evidence of pneumoperitoneum, only markedly dilated bowel loops with air–fluid levels. Consequently, on POD 10, she underwent re-exploration, and anastomotic leakage was found with dilated proximal bowel. A tapering jejunoplasty with reanastomosis was carried out manually, following which the child regained bowel movement by the POD 5 and was discharged on the POD 10. Unfortunately, the patient was lost to follow-up, and therefore, subsequent outcomes could not be assessed.

## Discussion

Intestinal atresia is categorized into five types based on the Grosfeld classification: Type I (mucosal web), Type II (fibrous cord atresia), Type IIIa (atresia with mesenteric defect), Type IIIb (apple peel or Christmas tree atresia), and Type IV (multiple atresias). 9 10 Type I atresia is most commonly found in the duodenum, and its occurrence in the jejunum is exceptionally rare, with the exact incidence remaining unknown. 4 Several theories have been proposed regarding the etiology of intestinal webs, including failure of recanalization, mesenteric vascular insult, disruption of endodermal development, and mucosal hyperproliferation. 6 11 It may be associated with other anomalies, such as gastroschisis and cystic fibrosis. 6 However, neither of our patients had any associated congenital anomalies.

Diagnosis becomes challenging when a central hole is present in the membrane, with the severity of symptoms depending on the location and aperture size. 11 While Type I jejunal atresia typically presents during the newborn period, cases with centrally perforated webs may present later, often being misdiagnosed as other causes of partial intestinal obstruction, such as malrotation, intestinal atresia, severe sepsis, and functional gastrointestinal obstruction. 7 12 In our series, both patients were initially misdiagnosed. In the first case, symptoms began at 7 months of age, but local physicians focused primarily on constipation, overlooking the episodes of bilious vomiting. The second case was even more difficult to recognize as surgical, given the long-standing history of non-bilious vomiting and severe malnutrition associated with dietary issues. She was managed symptomatically for an extended period and was only referred to a pediatric surgeon after the onset of bilious vomiting.

To establish the diagnosis, an upper gastrointestinal contrast study is the initial investigation following admission. 5 Contrast imaging typically demonstrates persistent dilation of the stomach and proximal intestine, with delayed passage of contrast on subsequent films. 11 Contrast studies may fail to detect thin webs, as contrast tends to pool on either side, and their visualization is possible only when the X-ray beam aligns parallel to the membrane. 4 13 Both of our patients underwent upper GI contrast films, and demonstrated dilatation of the proximal intestine but failed to reveal underlying pathology. Other investigations described in the literature for its diagnosis include ultrasonography, gastrointestinal endoscopy, CT scan, MRI, and video capsule endoscopy. 3 However, these investigations fail to serve as a definitive diagnostic tool for intestinal webs, as the diagnosis cannot be ruled out even when imaging studies yield negative results. 4 Definitive diagnosis of an intestinal web is established intraoperatively. Previous reports have mentioned performing a saline test after identification of a membrane to exclude additional distal webs. 8 12 Similarly, in our series, a saline test was performed in both cases; in the second patient, this facilitated identification of an additional membrane that might otherwise have been missed.

A recent review by Wong et al highlighted the scarcity of reports on jejunal atresias, with most cases managed by web excision. 6 However, some authors opted for resection and anastomosis—either end-to-end or side-to-side in cases with a markedly dilated proximal segment. 1 4 14 15 In our series, one patient underwent web excision while the other, who had double webs, required segmental resection and anastomosis. While enterotomy with web excision reduces the risk of leakage due to fewer sutures and by maintaining natural alignment, resection, and anastomosis of the ectatic bowel, though associated with a higher risk of leakage, help restore effective peristalsis and also secures the anastomosis by minimizing the luminal diameter discrepancy. Among the two previously reported cases of double jejunal web in English literature, one was managed with segmental resection, while the other underwent web excision. 7 8 Previous studies have shown no significant difference in outcomes between patients managed with web excision and those undergoing resection with anastomosis. 15

In the second case, tapering jejunoplasty was initially avoided to minimize suture lines and reduce the risk of leakage in a malnourished patient. However, during reoperation, jejunoplasty was the preferred approach to preserve the remaining bowel length. Wong et al also performed tapering jejunoplasty after en bloc resection of the atretic part. 6

Surgical excision remains the mainstay of treatment for jejunal webs, though various approaches have been described, including endoscopic laser therapy and combined laparotomy with endoscopy. 11 Most cases of jejunal web with delayed presentation recover well postoperatively, except for a few who had postoperative complications, such as anastomotic leakage, postoperative ileus, and sepsis. 3 Our patient with double webs had a complicated postoperative course due to anastomotic disruption. However, all the reported cases of delayed presentation of jejunal web were discharged from the hospital with full recovery. 1 3

## Conclusion

Delayed presentation of jejunal web with central fenestration, though rare, should be considered in cases of chronic vomiting and failure to thrive. Timely diagnosis with appropriate imaging and surgical intervention, whether by web excision or resection, typically leads to good outcomes.

## Notes

Importance for the Pediatric Surgeon Jejunal webs with central apertures may present beyond the neonatal period with subtle, non-specific symptoms, leading to diagnostic delays. Awareness of this rare entity is crucial for pediatric surgeons to ensure timely diagnosis and appropriate surgical management.
